# Mitochondria-endoplasmic reticulum contacts in sepsis-induced myocardial dysfunction

**DOI:** 10.3389/fcell.2022.1036225

**Published:** 2022-11-24

**Authors:** Tao Jiang, Qian Wang, Jiagao Lv, Li Lin

**Affiliations:** ^1^ Department of Geriatrics, Tongji Hospital, Tongji Medical College, Huazhong University of Science and Technology, Wuhan, China; ^2^ Division of Cardiology, Department of Internal Medicine, Tongji Hospital, Tongji Medical College, Huazhong University of Science and Technology, Wuhan, China

**Keywords:** sepsis-induced myocardial dysfunction, calcium signaling, autophagy, mitochondrial dynamics, ER stress, mitochondria-ER contacts, inflammation

## Abstract

Mitochondrial and endoplasmic reticulum (ER) are important intracellular organelles. The sites that mitochondrial and ER are closely related in structure and function are called Mitochondria-ER contacts (MERCs). MERCs are involved in a variety of biological processes, including calcium signaling, lipid synthesis and transport, autophagy, mitochondrial dynamics, ER stress, and inflammation. Sepsis-induced myocardial dysfunction (SIMD) is a vital organ damage caused by sepsis, which is closely associated with mitochondrial and ER dysfunction. Growing evidence strongly supports the role of MERCs in the pathogenesis of SIMD. In this review, we summarize the biological functions of MERCs and the roles of MERCs proteins in SIMD.

## 1 Introduction

Sepsis is defined as organ dysfunction due to the abnormal host immune response to infection (Singer et al., 2016). There are about 50 million patients worldwide diagnosed with sepsis (DeMerle et al., 2021); approximately half of septic patients develop cardiac dysfunction, which is referred as sepsis-induced myocardial dysfunction (SIMD) (Frencken et al., 2018). Mitochondria and endoplasmic reticulum (ER) are both important organelles in eukaryotic cells. Mitochondria are associated with important cellular biological processes such as energy conversion, redox equilibrium, calcium homeostasis, and so on. ER is the “protein and lipid synthesis base station” and it is related with the synthesis, modification, and processing of protein and lipid, as well as the regulation of calcium homeostasis (Li et al., 2019a; Govindarajan et al., 2020). Recently, it has shown that mitochondria and ER are closely related structurally and functionally, forming a unique structure referred to as mitochondria-ER contacts (MERCs) (Tabara et al., 2022), also called mitochondria-associated membranes (MAMs) (Pinton, 2018). MERCs are functionally involved in calcium signaling, lipid synthesis and transport, autophagy, mitochondrial dynamics, ER stress, and inflammation (Giorgi et al., 2015; Gao et al., 2020). Numerous studies have shown that SIMD has complex pathogenic mechanisms, including the release of circulating myocardial depressant factor, abnormal calcium signaling, mitochondrial dysfunction, and reactive oxygen species (ROS) release (Hollenberg and Singer, 2021), which are closely related to the biological functions of MERCs. With the deepening of research, an increasing number of studies indicates that MERCs play a crucial role in the occurrence and development of SIMD. This review will focus on the biological functions of MERCs and the roles of MERCs proteins in SIMD.

## 2 Discovery of MERCs

MERCs consist of mitochondrial outer membrane (MOM) and ER membrane, promoting the communication between mitochondria and ER. In 1956, Copeland and Dalton first described the contact between mitochondria and ER in pseudobranch cells (Copeland and Dalton, 1959). With the development of electron microscopy, it became possible to study the subtle structure of cells. In 1969, Ruby et al. Ruby et al. (1969) discovered the physical connection between MOM and ER using philips electron microscopy. In 1973, the crude extracts containing ER-mitochondria contact sites were isolated for the first time from liver by Lewis et al. Lewis and Tata. (1973). In 1990, Vance et al. Vance. (1990) isolated “fraction X″ from mitochondria, which had the activity of phospholipid synthesis and was later named MERCs (Vance, 2014). In 1999, Achleitner et al. Achleitner et al. (1999) revealed that the distance between ER and mitochondria in MERCs was found to be about 10–60 nm in yeast. In 2006, Csordás et al. Csordas et al. (2006) showed that the distance between the two organelles in MERCs was approximately 10–25 nm in rat liver. In 2009, some methods for extracting MERCs were proposed by Wiechowski et al. Wieckowski et al. (2009), allowing for further insight into the structure and function of MERCs. At present, there has been an increase of studies on MERCs, and more and more MERCs proteins have been identified. Zhang et al. Zhang et al. (2011) isolated MERCs using Percoll gradient fractionation and performed the first proteomic analysis of MERCs in human foreskin fibroblasts. In their work, there were 991 proteins were found. Poston and colleagues further isolated MERCs in mouse liver and brain, in which 961 and 1,212 proteins were identified by LC-MS/MS, respectively (Poston et al., 2013). As opposed to the previous approach, Hung et al. Hung et al. (2017) used engineered monomeric peroxidase APEX2 to target the ER membrane and mitochondrial outer membrane of HEK 293T cells, then they used SILAC mass spectrometry analysis to identify 94 potentially MERCs proteins. Comparing with Poston’s study, Ma et al. Ma et al. (2017) and Wang et al. Wang et al. (2018) identified more MERCs proteins in the mouse brain. This disparity may be caused by the difference in sample processing strategy, method and program setting of LC-MS/MS as well as the age and genetic background of the mouse strains. In addition to mouse brains, Wang et al. Wang et al. (2018) also performed proteomics on MERCs in human and mouse testis. Xue et al. (Lu et al., 2022) supplemented the data on skeletal muscle and myocardial tissue. In the next part, we will introduce the biological functions of MERCs.

## 3 Biological functions of MERCs

**FIGURE 1:**
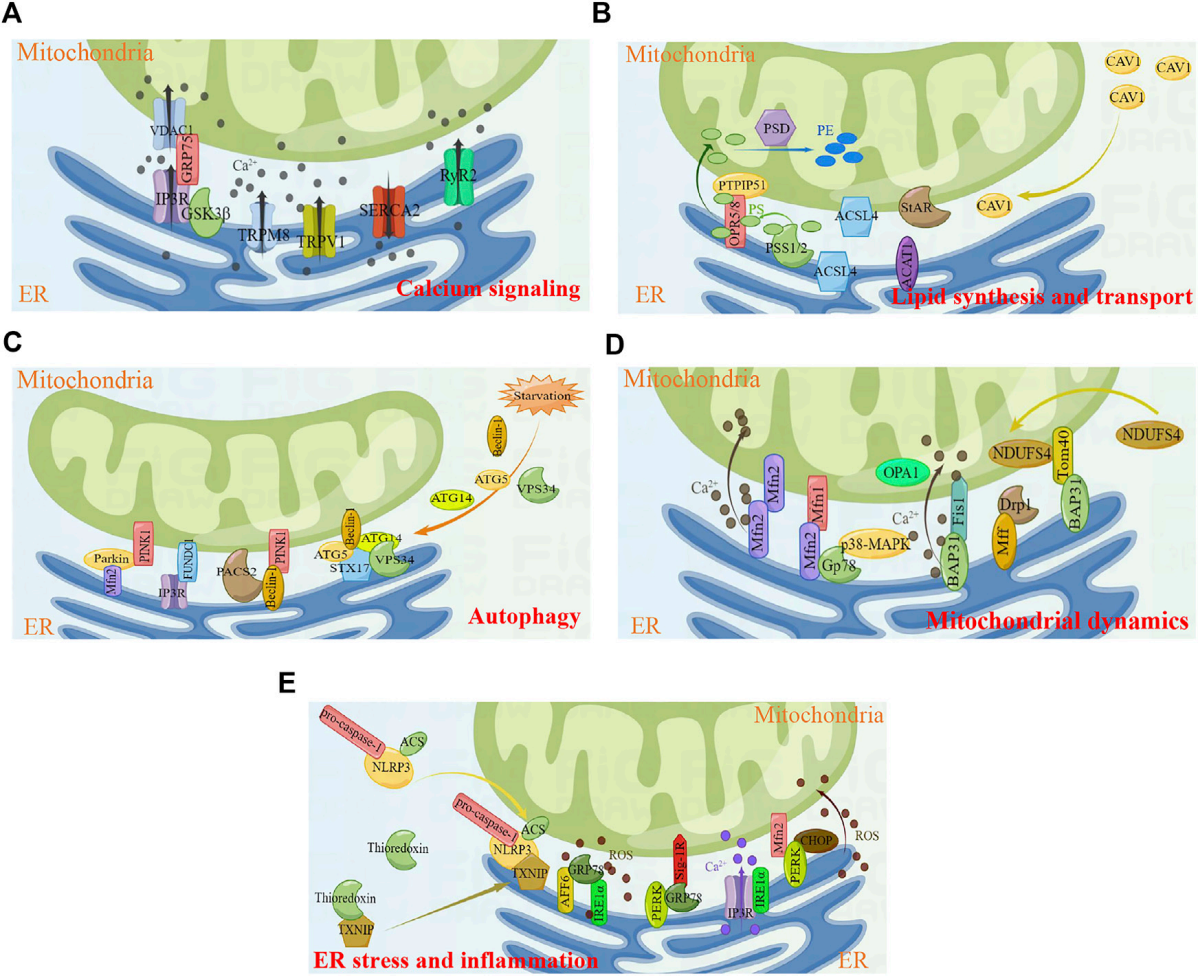
Key cellular functions and proteins involved in MERCs. **(A)** Calcium signaling. **(B)** Lipid synthesis and transport. **(C)** Autophagy. **(D)** Mitochondrial dynamics. **(E)** ER stress and inflammtion. Figures were created using Figdraw. ER, endoplasmic reticulum; MERCs, mitochondria-endoplasmic reticulum contacts.

MERCs are involved in several cellular biological processes, including calcium signaling, lipid synthesis and transport, autophagy, mitochondrial dynamics, and ER stress (Figure 1). Thus, MERCs have received extensive attention in recent years.

### 3.1 Calcium signaling

Calcium homeostasis is critical for several cellular activities, and abnormal calcium handling will contribute to cellular dysfunction, which in turn leads to cell death. Calcium homeostasis is associated with the absorption and release of Ca^2+^. Ca^2+^ is usually stored in ER, and Ca^2+^ will release from ER to the cytoplasm when cells are stimulated. In addition, a small amount of Ca^2+^ can enter the cell from extracellular milieu through plasma membrane Ca^2+^ channels. The cytoplasmic Ca^2+^ can be absorbed by mitochondria to regulate intracellular calcium balance and mitochondrial energy metabolism. MERCs, the overlapping regions between ER and mitochondria, are identified as ‘hotspots’ and have pivotal roles in the highly efficient transmission of Ca^2+^ (Patergnani et al., 2011).

The transport of Ca^2+^ is regulated by numerous MERCs proteins. Inositol 1, 4, 5-triphosphate receptor (IP3R) is a well-established ER-resident Ca^2+^ channel. IP3 binds to IP3R, triggering the opening of the IP3R channel and allowing the release of Ca^2+^ from ER into the cytoplasm (Lee et al., 2021). Voltage-dependent anion-selective channel protein 1 (VDAC1) is an ion channel localized on the MOM, regulating mitochondrial Ca^2+^ uptake (Shoshan-Barmatz and Golan, 2012). IP3R indirectly interacts with VDAC1 through glucose-regulated protein 75 (GRP75), forming the IP3R/GRP75/VDAC1 complex in MERCs and mediating ER/mitochondria Ca^2+^ exchange (Benade et al., 2020). The IP3R/GRP75/VDAC1 complex further interacts with Sig-1R to regulate MERCs formation. Naia et al. reveal that pridopidine, a selective Sig-1R agonist, enhances mitochondria-ER association (Naia et al., 2021). In addition, a previous study has shown that IRBIT is also involved in the stability of MERCs, which may be related to the interaction between IP3R and IRBIT (Bonneau et al., 2016). Ryanodine receptor type 2 (RyR2) and sarcoplasmic/endoplasmic reticulum calcium ATPase 2 (SERCA2) are also important Ca^2+^ release and Ca^2+^ uptake channels in MERCs, respectively; they play a key role in myocardial contraction and relaxation (Conesa et al., 2020). During cardiac excitation–contraction coupling, a minor amount of extracellular Ca^2+^ enters the cytoplasm through L-type voltage gated calcium channels, triggering the release of large amounts of Ca^2+^ into the cytoplasm through RyR2 channels and causing cardiomyocyte contraction. Upon completion of the cardiomyocyte contraction, most of the cytoplasmic Ca^2+^ is reabsorbed into ER *via* SERCA2 (Tester et al., 2020). Apart from the RyR2 protein level, the function of RyR2 is also regulated by its phosphorylation at Ser 2,808 (Baine et al., 2020; Potenza et al., 2020). Glycogen synthase kinase 3β (GSK‐3β) is a kind of multifunctional kinase. Studies have found that part of GSK3β is localized on MERCs and interacts with the IP3R/GRP75/VDAC1 complex. Pharmacological and genetic inhibition of GSK3β attenuates the interaction between IP3R and VDAC1/GRP75, thereby impairing ER Ca^2+^ release and ER/mitochondria Ca^2+^ exchange (Gomez et al., 2016). Recently, transient receptor potential M8 (TRPM8) has been identified as a functional Ca^2+^ release channel localized on MERCs (Izquierdo et al., 2021). The study by Xiong et al. find that TRPM8 is involved in the regulation of mitochondrial calcium homeostasis in vascular smooth muscle cells. Activation of TRPM8 antagonizes angiotensin II-induced mitochondrial respiratory dysfunction and excess ROS production by maintaining mitochondrial Ca^2+^-dependent pyruvate dehydrogenase activity, which in turn inhibits cold or angiotensin II-induced elevated blood pressure in mice (Xiong et al., 2017). Transient receptor potential vanilloid 1 (TRPV1) is a non-selective cation channel that can be activated by different physical and chemical stimuli. Activation of TRPV1 increases the mitochondrial Ca^2+^ level and leads to mitochondrial depolarization (Wang et al., 2021a; Li et al., 2021). Wei et al. Wei et al. (2020) reveals that activation of TRPV1 channel by capsaicin attenuates hyperglycemia-induced mitochondrial dysfunction in podocytes *via* inhibiting MERCs formation and reducing Ca^2+^ transport from the ER into mitochondria.

### 3.2 Lipid synthesis and transport

Lipids are important components of cell membranes and are involved in energy storage, transduction of the signaling molecules, and synthesis of active substances. Lipid synthesis usually occurs in the ER, whereas mitochondria are associated with lipid modifications. MERCs are involved in lipid biosynthesis and lipid transport between the ER and mitochondria.

Phosphatidylserine (PS) is a glycerophospholipid resided in the cytoplasm (Malek et al., 2021). PS is synthesized by phosphatidylserine synthase 1 (PSS1) and phosphatidylserine synthase 2 (PSS2), which are localized on MERCs (Kuge and Nishijima, 1997; Vance, 2014). PS binds to MERCs molecules ORP5/ORP8, followed by translocation to mitochondria *via* the MOM protein PTPIP51 (Galmes et al., 2016). After PS transfers to mitochondria, PS is converted to phosphatidylethanolamine (PE) *via* PE decarboxylase (PSD), a mitochondrial inner membrane protein (Voss et al., 2012). Long chain acyl-CoA synthetase 4 (ACSL4), one of the key enzymes in lipid synthesis, is reported to be enriched on MERCs (Radif et al., 2018). In addition, MERCs also provide enzymes related to cholesterol metabolism and transport, including ACAT1, StAR, and so on (Rusinol et al., 1994; Prasad et al., 2015). Caveolin-1 (CAV1), the major structural protein of caveolae, is associated with the regulation of hepatic lipid storage and trafficking (Han et al., 2020) as well as transmembrane transport of long chain fatty acids (Meshulam et al., 2006). Overexpression of CAV1 in hepatocytes stimulates cholesterol efflux through promoting the transfer of cholesterol to cholesterol-rich regions in the plasma membrane (Fu et al., 2004). In addition, Zhang et al. Zhang et al. (2020a) find that CAV1–deficient mice attenuate the atherosclerosis. Sala-Vila et al. (Sala-Vila et al., 2016) isolate highly purified MERCs fractions from mouse liver and perform in-depth mass spectrometry. The results showed that CAV1 was enriched in MERCs and is associated with mitochondrial function, lipid balance and metabolic homeostasis. The livers of CAV1-knockout mice exhibit reduced MERCs physical extension and abnormal accumulation of free cholesterol.

### 3.3 Autophagy

Autophagy is an important cellular mechanism, in which autophagosome encapsulates degraded proteins, as well as aged or damaged organelles, and then fuses with lysosomes to degrade the components in the vesicles. Autophagy plays an important role in a variety of physiological and pathological processes, including cell differentiation and development, regulation of growth and aging, and clearance of damaged or aging organelles (Cui et al., 2020).

Many proteins localized on MERCs are involved in autophagy, including Beclin-1, ATG5, ATG14, VPS34, PINK1, Parkin, FUNDC1, etc. MERCs are important sites for autophagosome formation, and autophagy proteins Beclin-1, ATG5, ATG14, and VPS34 will be recruited to MERCs when cells are stimulated by starvation (Hamasaki et al., 2013). Beclin-1 is a highly conserved autophagy regulator protein in eukaryotic cells, which is mainly responsible for inducing the nucleation of autophagosomes during autophagy. Overexpression of Beclin-1 enhances autophagy and reduces apoptosis (Chang et al., 2021). It has been found that Beclin-1 relocates to MERCs and interacts with PINK1 to promote the formation of MERCs and autophagosome precursor in mitophagy and starvation-induced autophagy (Gelmetti et al., 2017). A diabetic nephropathy study reveals that phosphofurin acidic cluster sorting protein 2 (PACS2) binds to Beclin-1 and mediates the reorientation of Beclin-1 to MERCs, followed by the formation of mitophagosome (Li et al., 2022). In addition to being involved in the initiation of autophagosome, ATG14 also interacts with MERCs protein syntaxin 17 (STX17) to promote autophagosome maturation (Manganelli et al., 2021).

Autophagy initiated by Beclin-1, ATG5, ATG14, and VPS34 is the non-selective autophagy; in addition to this, there is also selective autophagy. Selective autophagy can specifically degrade different cellular components, such as mitochondria, ER, ribosomes, and so on (Makino et al., 2021). The selective autophagy of mitochondria is referred to mitophagy, which selectively removes damaged or aging mitochondria and is the important mechanism of mitochondrial quality control. There are two major mitophagy pathways, including PINK1/Parkin-mediated mitophagy and mitochondrial receptor-mediated mitophagy. PINK1 is a serine/threonine kinase. In the physiological state, PINK1 enters the mitochondrial inner membrane through mitochondrial inner and outer membrane transporters, and is subsequently degraded by mitochondrial processing peptidases and presenilin-associated rhomboid-like protein. When the mitochondrial membrane potential is damaged due to radiation, ROS or chemotherapeutic drugs, PINK1 will stabilize on the MOM and phosphorylate Parkin at Ser 65 through its ubiquitin-like domain, followed by the initiation of mitophagy (Lazarou et al., 2015; Zheng et al., 2021). Findings by Gelmetti et al. (Gelmetti et al., 2017) show that PINK1 localizes on MERCs in physiological state, and after using CCCP to enhance mitophagy, the localization of PINK1 on MERCs increases. Conversely, silencing of PINK1 results in reduced MERCs formation and increased distances between organelles in MERCs (Parrado-Fernandez et al., 2018). Consistent with PINK1, Parkin is also confirmed to be involved in the formation of MERCs. It has been found that overexpression of Parkin significantly increases the formation of MERCs and promotes Ca^2+^ transport and mitochondrial ATP production (Cali et al., 2013). Regarding the mechanism by which Parkin affects the formation of MERCs, a study by Basso et al. (Basso et al., 2018) reveals that it may be related to the ubiquitination of mitochondrial fusion protein 2 (Mfn2) mediated by Parkin, and non-ubiquitinable Mfn2 mutant cannot restore the structure and function of MERCs. FUN14 domain containing 1 (FUNDC1), a three-transmembrane protein localized on MOM, is a mammalian mitophagy receptor and contains the LC3-interacting region that interacts with LC3 to induce mitophagy (Cai et al., 2021). Recently, studies show that FUNDC1 interacts with IP3R to form a tethering complex in the MERCs region, which is associated with ER/mitochondria Ca^2+^ exchange and mitochondrial dynamics, leading to cardiac dysfunction and heart failure (Wu et al., 2017; Wu et al., 2019; Ren et al., 2020). In addition, the reduction of FUNDC1-dependent MERCs formation decreases vascular endothelial growth factor receptor 2 (VEGFR2) levels and inhibits angiogenesis (Wang et al., 2021b).

### 3.4 Mitochondrial dynamics

Mitochondrial dynamics refers to the process of mitochondrial fission and fusion; the balance between the two opposing processes is critical for maintaining mitochondrial number, morphology, and size, as well as cell survival (Wang et al., 2020a; Brzoskwinia et al., 2021).

Dynamin-related protein 1 (Drp1) and mitochondrial fission factor (Mff) are both localized on MERCs to function (Cherubini et al., 2020; Luan et al., 2021; Zhong et al., 2022). Drp1 is the important effector molecule in mitochondrial fission. It is a cytoplasmic GTPase that is recruited from the cytoplasm to mitochondria and forms contractile ring, thereby driving the cutting process of mitochondria (Hu et al., 2020). The recruitment of Drp1 from the cytoplasm to mitochondria requires the assistance of MOM protein Mff, and overexpression of Mff promotes mitochondrial fission (Wang et al., 2020b). On the other hand, mitochondrial dynamics is regulated by mitochondrial fusion protein 1 (Mfn1), Mfn2, and optic atrophy 1 (OPA1). Mfn2, a GTPase localizes on the ER, dimerizes with Mfn1 or Mfn2 on the MOM to drive mitochondrial fusion (de Brito and Scorrano, 2008; Dorn et al., 2015). This interaction modulates ER and mitochondrial dynamics; however, this widely accepted model is challenged by the quantitative analysis of MERCs. One study shows that knock-down of Mfn2 increases intimate contact between ER and mitochondria, and then promotes Ca^2+^ transfer from the ER to mitochondria (Dentoni et al., 2022a). Here, Mfn2 acts more like as a repressor of mitochondrial and ER contact, which prevents excessive proximity of the two organelles. Therefore, the exact role of Mfn2 in MERCs remains controversial, and more studies are required to elucidate this. Gp78, an E3 ubiquitin ligase localized on the ER, induces the degradation of Mfn1 and Mfn2. Gp78 phosphorylation at Ser 538 induced by p38 MAPK decreases the capacity for degradation of Mfn1 and Mfn2, influencing MERCs formation and mitochondrial dynamics (Li et al., 2015).

Another tethering complex of MERCs is the ER-resident B-cell receptor-associated protein 31 (BCAP31) and MOM protein Fis1 that initiates the mitochondrial fission process (Horibata et al., 2020). BAP31 interacts with Fis1 to induce mitochondrial fission, leading to mitochondrial damage (Cheng et al., 2021). In addition, the interaction of Fis1 and BAP31 cleaves BAP31 into p20 and transmits pro-apoptotic signal from mitochondria to ER, which in turn triggers the transfer of Ca^2+^ from the ER to mitochondria, decreases mitochondrial membrane potential, and initiates cell death (Cui et al., 2020). BAP31 also interacts with mitochondrial protein Tom40 to induce the translocation of mitochondrial respiratory chain complex I subunit NDUFS4 from cytoplasm to mitochondria, which is essential for maintaining mitochondrial function. Following loss of BAP31 function, cells develop impaired cellular metabolism, activation of AMPK signaling, and reduced mitochondrial oxygen consumption–dependent ATP levels (Namba, 2019).

### 3.5 ER stress and inflammation

ER is involved in the synthesis, folding, and modification of secreted and transmembrane proteins, and this process is tightly regulated. However, there are still a variety external and internal events that disrupt the folding capacity of ER proteins and trigger the accumulation of misfolding or unfolded proteins, namely ER stress (Chen and Cubillos-Ruiz, 2021).

In mammalian cells, three ER transmembrane proteins exist as ER stress sensors, namely activating transcription factor 6 (ATF6), inositol-requiring enzyme 1α (IRE1α), and protein kinase R-like ER kinase (PERK). When cells are in the steady state, glucose-regulated protein 78 (GRP78) binds to the aforementioned ER stress sensors to keep them in an inactive state; when cells are under stress, GRP78 shows a higher affinity for misfolded or unfolded proteins, leading to the activation of ER stress sensors and unfold protein response (i.e., ER stress) (Alsterda et al., 2021). Sig-1R forms a complex with GRP78 and localized to MERCs, and Sig-1R will dissociates from GRP78 due to ER Ca^2+^ depletion or ligand stimulation, thereby affecting Ca^2+^ transport (Hayashi and Su, 2007). Carreras-Sureda et al. Carreras-Sureda et al. (2019) find that IRE1α is involved in maintaining the structure and function of MERCs under resting conditions, mainly by forming protein complexes with IP3R and affecting the localization of IP3R on MERCs and its channel activity. PERK is enriched in MERCs and interacts with Mfn2 to maintain the structure and function of MERCs. In ROS-mediated ER stress, PERK promotes the transmission of ROS signals between the ER and mitochondria and apoptosis by maintaining the level of C/EBP homologous protein (CHOP) and the function of MERCs (Fan and Jordan, 2022). Zhang et al. Zhang et al. (2020b) find that skeletal muscle of type 2 diabetic mice exhibits reduced MERCs formation, impaired mitochondrial mass, and enhanced ER stress, which are significantly associated with ATF6.

NOD-like receptor pyrin domain-containing 3 (NLRP3) inflammasome, including NLRP3, C-terminal caspase recruitment domain (ASC), and pro-caspase-1, is currently reported to be associated with MERCs. In the resting state, NLRP3 is located in cytoplasm and ER, and ASC is mostly located in cytoplasm. Upon stimulation, NLRP3 and ASC are redistributed to the perinuclear, where they co-locate to MERCs to sense mitochondrial damage (Zhou et al., 2011). In resting cells, thioredoxin-interacting protein (TXNIP), a protein linked to insulin resistance, TXNIP interacts with thioredoxin. Zhou et al. Zhou et al. (2010) find that the inflammasome activator could induce TXNIP to dissociate from thioredoxin and bind to NLRP3. In addition, TXNIP deficiency impairs activation of NLRP3 inflammasome. Bronner and colleagues find that *brucella* infection causes NLRP3 inflammasome activation, which promotes TXNIP localization to MERCs (Bronner et al., 2015). Therefore, MERCs play a key role in initiating inflammation as an inflammatory platform.

## 4 The role of MERCs in SIMD

**FIGURE 2:**
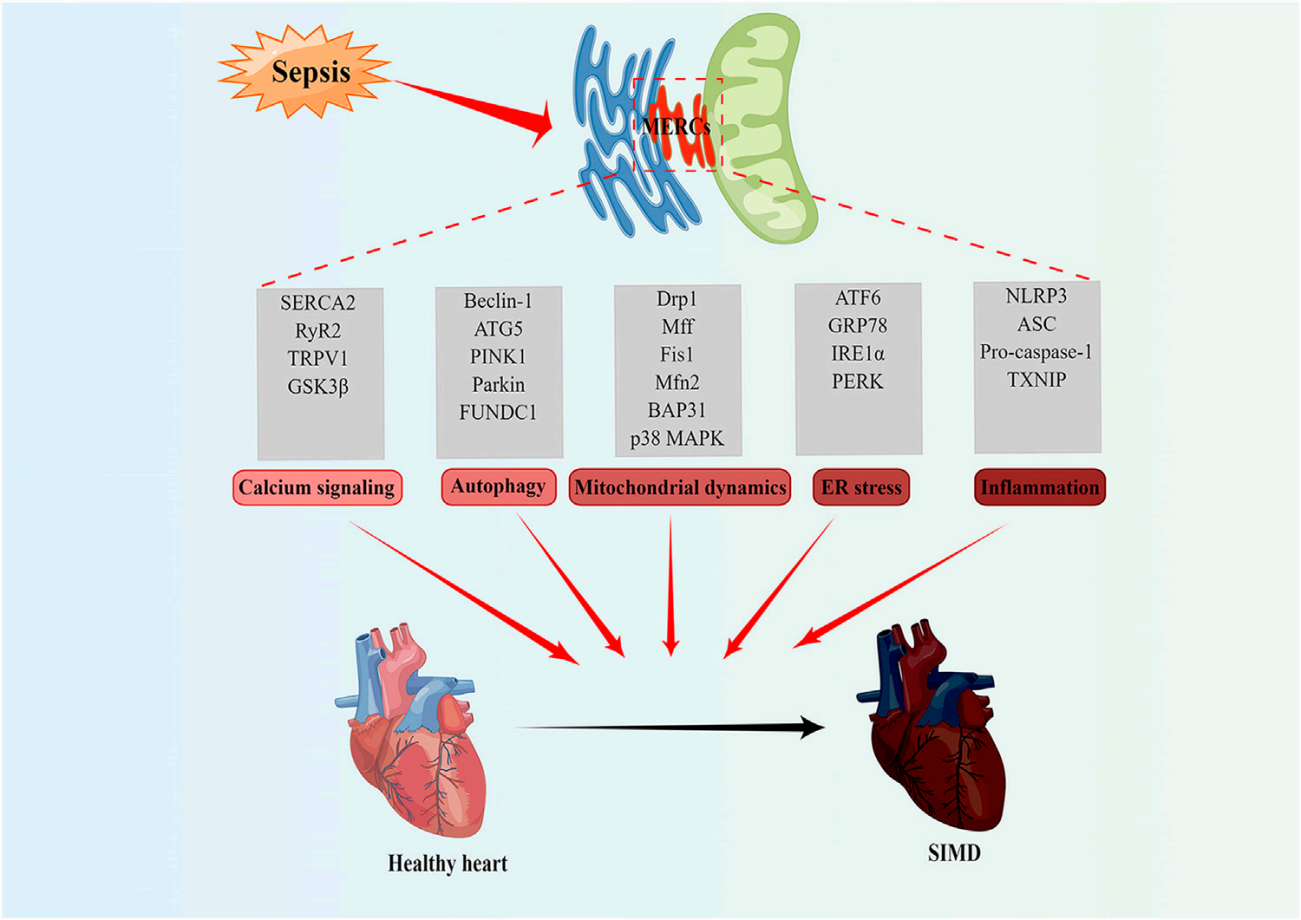
MERCs as potential new therapeutic targets for treatment of SIMD. MERCs regulates some important biological functions, including calcium signaling, autophagy, mitochondrial dynamics, ER stress, and inflammation. The abnormality of these processes often leads to SIMD. Notably, the key regulatory proteins of these processes can serve as potential therapeutic targets for SIMD. Figures were created using Figdraw. ER, endoplasmic reticulum; MERCs, mitochondria-endoplasmic reticulum contacts; SIMD, sepsis-induced myocardial dysfunction.

Abnormal mitochondrial and endoplasmic reticulum functions are important pathogenic mechanisms of SIMD. A large number of studies have found that MERCs proteins play an important role in SIMD through affecting calcium signaling, autophagy, mitochondrial dynamics and ER stress (Table 1; Figure 2).

**TABLE 1:** Components of MERCs involved in SIMD.

Proteins	Alterations	Study models	References
Calcium signaling			
SERCA2	SERCA oxidation ↑	LPS-treated mice; LPS-treated primary cardiomyocytes	Pang et al. (2019)
	sulfonylation of SERCA (Cys674) ↑	LPS-treated mice	Hobai et al. (2013)
	SERCA dysfunction	CLP mice	Luptak et al. (2019)
	SERCA2 ↓	LPS-treated left atrial myocytes	Tai et al. (2019)
	SERCA ↓	LPS-treated mice	Saiyang et al. (2021)
RyR2	Phosphorylation of RyR2 (Ser2808) ↑	LPS-treated left atrial myocytes	Tai et al. (2019)
	RyR2 ↓	LPS-treated mice	Saiyang et al. (2021)
TRPV1	Internalization of TRPV1 ↑	LPS-treated hiPSC-CM	Sattler et al. (2021)
GSK3β	GSK-3β ↑	LPS-treated rats	He et al. (2015)
	GSK-3β ↑	LPS-treated rat; LPS-treated primary cardiomyocytes	Li et al. (2019b)
	Phosphorylation of GSK-3β ↓	CLP rats	Jin et al. (2020)
Autophagy			
Beclin-1	Beclin-1 ↑	LPS-treated H9c2 cells	Lei et al. (2018)
	Beclin-1 ↓	LPS-treated mice	Sun et al. (2018)
	Beclin-1 ↓	LPS-treated mice	Sun et al. (2021)
	Acetylation of Beclin-1 ↑	CLP rats	Pi et al. (2021)
ATG5	ATG5 ↓	CLP mice	Wang et al. (2016)
	ATG5 ↑	LPS-treated primary cardiomyocytes	Wang et al. (2014)
PINK1/Parkin	Parkin ↓	LPS-treated mice; LPS-treated primary cardiomyocytes	Shang et al. (2020)
	Parkin ↓	LPS-treated mice; LPS-treated HL-1 cells	Cao et al. (2020)
	Parkin ↓	LPS-treated mice	Essandoh et al. (2019)
	Parkin ↓	LPS-treated mice	Piquereau et al. (2013)
	Parkin ↓	LPS-treated H9c2 cells	Bin et al. (2021)
	PINK1/Parkin ↑	LPS-treated mice	Ji et al. (2021)
	PINK1/Parkin ↑	CLP mice	Rahim et al. (2021)
	PINK1 ↑	LPS-treated mice; LPS-treated H9c2 cells	Jiang et al. (2021a)
FUNDC1	FUNDC1 ↓	LPS-treated H9c2 cells	Jiang et al. (2021b)
	FUNDC1 ↓	LPS-treated mice	Wang et al. (2021c)
	FUNDC1 ↑	LPS-treated mice; LPS-treated H9c2 cells	Jiang et al. (2021a)
	FUNDC1 ↑	LPS-treated mice	Ji et al. (2021)
Mitochondrial dynamics			
Drp1	Drp1 ↑	LPS-treated H9c2 cells	Tian et al. (2020)
	Drp1 ↑	LPS-treated mice	Yu et al. (2019)
	Interaction of Drp1 and Fis1 ↑	LPS-treated mice; LPS-treated H9c2 cells	Haileselassie et al. (2019)
	Drp1 ↑	CLP mice	Rahim et al. (2021)
	Drp1 ↓	LPS-treated H9c2 cells	Li et al. (2019c)
Mff	Mff ↑	LPS-treated H9c2 cells	Tian et al. (2020)
Fis1	Fis1 ↑	LPS-treated H9c2 cells	Tian et al. (2020)
	Interaction of Drp1 and Fis1 ↑	LPS-treated mice; LPS-treated H9c2 cells	Haileselassie et al. (2019)
Mfn2	Mfn2 ↓	CLP mice	Rahim et al. (2021)
BAP31	BAP31 ↓	LPS-treated mice; LPS-treated primary cardiomyocytes	Zhang et al. (2020c)
p38 MAPK	Phosphorylation of p38-MAPK ↑	LPS-treated mice	Wang et al. (2021d)
	Phosphorylation of p38-MAPK ↑	LPS-treated H9c2 cells	Shyni et al. (2021)
	Phosphorylation of p38-MAPK ↑	LPS-treated H9c2 cells	Rocca et al. (2021)
ER stress			
ATF6	ATF6 ↑	CLP rats; LPS-treated cardiomyocytes	Xia et al. (2022)
	ATF6 ↑	CLP mice	Han et al. (2017)
GRP78	GRP78 ↑	CLP mice	Xu et al. (2018)
	GRP78 ↑	CLP mice	Han et al. (2017)
	GRP78 ↑	LPS-treated mice; LPS-treated primary cardiomyocytes	Ceylan-Isik et al. (2010)
	GRP78 ↑	LPS-treated HL-1 cells	Zou et al. (2014)
	GRP78 ↓	LPS-treated rats	Chen et al. (2020)
IRE1α	Phosphorylation of IRE1α (Ser724) ↑	CLP rats; LPS-treated cardiomyocytes	Xia et al. (2022)
	IRE1α ↑	CLP mice	Han et al. (2017)
	IRE1α ↑	LPS-treated mice; LPS-treated primary cardiomyocytes	Ceylan-Isik et al. (2010)
	Phosphorylation of IRE1 ↑	LPS-treated HL-1 cells	Zou et al. (2014)
PERK	PERK ↑	LPS-treated mice; LPS-treated primary cardiomyocytes	Ceylan-Isik et al. (2010)
	Phosphorylation of PERK ↑	LPS-treated HL-1 cells	Zou et al. (2014)
Inflammation			
NLRP3	NLRP3 ↑	LPS-treated H9c2 cell	Qiu et al. (2019)
	Translocation of NLRP3 ↑	LPS-treated mice; LPS-treated H9c2 cell	Li et al. (2019d)
	NLRP3 ↑	CLP mice	Busch et al. (2021)
ASC	ACS ↑	LPS-treated H9c2 cell	Qiu et al. (2019)
Pro-caspase-1	Pro-caspase-1 ↑	LPS-treated H9c2 cell	Qiu et al. (2019)
TXNIP	Dissociation of TXNIP from thioredoxin ↑	LPS-treated mice; LPS-treated H9c2 cell	Li et al. (2019d)
	TXNIP ↑	LPS-treated H9c2 cell	Yang et al. (2019)

Abbreviations: CLP, cecal ligation and puncture; hiPSC-CM, human induced pluripotent stem cell-derived cardiomyocytes; LPS, lipopolysaccharide; MERCs, mitochondria-endoplasmic reticulum contacts; SIMD, sepsis-induced myocardial dysfunction; ↑ indicates an increase; ↓ indicates a decrease.

### 4.1 MERCs-associated calcium signaling in SIMD

SIMD impairs cardiac systolic and diastolic function, and in lipopolysaccharide (LPS)-induced sepsis, mice exhibit the abnormalities of cardiac ultrasonography and ultrastructure, as well as SERCA oxidation and disturbance of intracellular calcium (Pang et al., 2019). Hobai et al. Hobai et al. (2013) reveal that LPS contributes to cardiac dysfunction *via* sulphonylation of SERCA Cys 674 and inhibition of Ca^2+^ influx. Consistent with this, mice that suffer from cecal ligation and puncture (CLP) develop cardiomyocyte sarcomere shortening, inhibition of Ca^2+^ transients, and SERCA dysfunction (Luptak et al., 2019). Tai et al. Tai et al. (2019) utilize LPS to stimulate left atrial cardiomyocytes and find that LPS results in a significant decrease in SERCA2 expression and an increase in RyR2 phosphorylation. Xie et al. (Saiyang et al., 2021) find that loxoribine, the toll-like receptor 7 (TLR7) agonist, ameliorates LPS-induced cardiac dysfunction, which is associated with the upregulation of p-PLN (Ser 16) *via* cAMP-PKA pathway, thereby promoting the expression of SERCA and RyR2 and ultimately restoring the abnormal Ca^2+^ handling. A recent study reveals that TRPV1 is also altered in LPS-treated cardiomyocytes. In LPS-treated human-induced pluripotent stem cell-derived cardiomyocytes, TRPV1 channels internalize and their mediated ion channel currents are significantly reduced (Sattler et al., 2021). He et al. He et al. (2015) find that septic rats induced by LPS develop cardiomyocytes swelling, degeneration, and loss of transverse striations, accompanied by a significant increase in GSK-3β levels. Further study shows that inhibition of GSK-3β attenuated LPS-induced myocardial injury by inhibiting FOXO3A activation (Li et al., 2019b). However, it is found in the CLP-induced myocardial injury model that decrease in p-GSK-3β/GSK-3β ratio promotes inflammation and myocardial injury (Jin et al., 2020).

### 4.2 MERCs-associated autophagy in SIMD

LPS treatment results in cardiomyocyte death, lactate dehydrogenase release, and increased production of the lipid peroxidation product malondialdehyde, which may be related to autophagy activation caused by increased Beclin-1 levels (Lei et al., 2018). Sun et al. Sun et al. (2018) apply Beclin-1 knockout and overexpression mice to find that Beclin-1 dependent autophagy improves cardiac mitochondrial function during sepsis, thereby improving cardiac function and circulating inflammation levels, and improving mouse survival. Further study finds that LPS leads to the impaired structure and function of cardiac MERCs, while cardiac-specific Beclin-1 overexpression restores the structure and function of MERCs and improves cardiac function in septic mice (Sun et al., 2021). In rat sepsis model induced by CLP, sepsis induces cardiac Beclin-1 acetylation to inhibit autophagy, resulting in impaired cardiac function. Treatment with melatonin promotes deacetylation of Beclin-1, leading to improved cardiac function (Pi et al., 2021). The above studies suggest that both Beclin-1 expression and post-expression modification play important roles in the occurrence and development of SIMD. In addition to Beclin-1, ATG5 has also been confirmed to be involved in the development of SIMD (Wang et al., 2016). However, the activation of autophagy may also aggravate SIMD. Wang et al. Wang et al. (2014) find that LPS induces myocardial injury, with upregulated ATG5 mRNA, Beclin-1 mRNA and LC3II protein levels, while estrogen protects cardiomyocytes *via* inhibiting autophagy. Therefore, the role of MERCs-related non-selective autophagy in SIMD remains to be further elucidated.

MERCs-associated mitophagy also plays an important role in SIMD. In the LPS-induced septic cardiomyopathy model, PINK1/Parkin-dependent mitophagy is significantly reduced, while mitochondrial homeostasis and cardiomyocyte activity are enhanced after increasing mitophagy by Mst1 deletion, which in turn is reversed by inhibition of Parkin-related mitophagy (Shang et al., 2020). Studies by Cao et al. Cao et al. (2020), Essandoh et al. Essandoh et al. (2019), and Piquereau et al. Piquereau et al. (2013) also support the proective effects of PINK1/Parkin-dependent mitophagy on cardiac function in mice with sepsis. In H9c2 cells, activation of the PINK1/Parkin pathway is also found to attenuate mitochondrial damage, oxidative stress, and apoptosis induced by LPS treatment (Bin et al., 2021). However, Ji et al. Ji et al. (2021) find that PINK1/Parkin-dependent mitophagy aggravates sepsis-induced myocardial injury. Rahim et al. Rahim et al. (2021) and Jiang et al. Jiang et al. (2021a) also show that the levels of PINK-1/Parkin signaling are greatly increased in sepsis-induced heart injury. The role of FUNDC1-dependent mitophagy in SIMD has also received extensive attention. LPS treatment significantly reduces FUNDC1-dependent mitophagy in H9c2 cardiomyocytes, resulting in mitochondrial dysfunction, oxidative stress, and apoptosis; irisin treatment attenuates LPS-induced cell damage by inhibiting FUNDC1-dependent mitophagy (Jiang et al., 2021b). Wang et al. Wang et al. (2021c) find that FUNDC1 knockout abolished the cardioprotective effect of mitophagy activators in septic mice. However, contrary to this, Jiang et al. Jiang et al. (2021a) reveal that LPS treatment increases the formation of MERCs, promotes intracellular Ca^2+^ overload and ROS production, activates mitophagy, and decreases mitochondrial membrane potential and intracellular ATP levels, which reversed by knockdown of FUNDC1. Another study also shows that the level of FUNDC1 is significantly increased in LPS-induced myocardial injury, and improvement in myocardial injury is accompanied by a decrease in FUDNC1 levels (Ji et al., 2021). In conclusion, MERCs-associated mitophagy plays an important role in SIMD, but its specific effects need to be further elucidated.

### 4.3 MERCs-associated mitochondrial dynamics in SIMD

Mitochondrial dynamics include mitochondrial fission and mitochondrial fusion, the balance of which is critical for maintaining mitochondrial structure and function. A study shows that LPS treated H9c2 cardiomyocytes develop mitochondrial fragmentation and increased mRNA levels of Drp1, Mff and Fis1 (Tian et al., 2020). Inhibition of Drp1-related mitochondrial fission attenuates septic cardiomyopathy induced by LPS (Yu et al., 2019). Haileselassie et al. Haileselassie et al. (2019) find that interfering with the interaction between Drp1 and Fis1 depresses cardiac mitochondrial fragmentation, alleviates cardiac dysfunction, and reduces mouse mortality. Rahim et al. Rahim et al. (2021) also find increased mitochondrial fission in cardiac tissues of CLP-induced sepsis mice, manifested as increased Drp1 content and decreased Mfn2 level; whereas melatonin improves SIMD prognosis by increasing Mfn2/Drp1 ratio. However, Li et al. Li et al. (2019c) find that use of Mdivi-1, a mitochondrial division inhibitor, aggravates LPS-induced apoptosis. In addition, LPS treatment results in a reduction of BAP31 levels in cardiomyocytes, while melatonin treatment restores BAP31 expression; BAP31 knockdown attenuates the beneficial effects of melatonin on mitochondrial function and ER homeostasis under LPS stress (Zhang et al., 2020c). Several studies have shown that p38 MAPK plays an essential role in SIMD, but the main focus of these studies is on its effect on the NF-κB inflammatory signaling pathway (Wang et al., 2021d; Rocca et al., 2021; Shyni et al., 2021), whether p38 MAPK plays a role in SIMD through the mechanism of Gp78 affecting the degradation of Mfn1 and Mfn2 deserves further investigation.

### 4.4 MERCs-associated ER stress in SIMD

The expressions of ATF6, GRP78 and IRE1α are significantly up-regulated in LPS-treated cardiomyocytes and heart tissues of CLP-induced sepsis model (Han et al., 2017; Xu et al., 2018; Xia et al., 2022). The study of Ceylan-Isik et al. Ceylan-Isik et al. (2010) also confirms that the levels of GRP78, IRE1α and PERK are significantly increased in LPS-induced murine septic cardiomyopathy. Consistent with this, LPS also results in increased levels of GRP78, phosphorylated IRE1, and phosphorylated PERK in murine atrial myocytes (Zou et al., 2014). However, Chen et al. Chen et al. (2020) find that the protein level of GRP78 is significantly decreased in the heart tissue of LPS-treated mice. These results support that MERCs-associated ER stress plays an important role in SIMD.

### 4.5 MERCs-associated inflammation in SIMD

The key role of inflammation in the pathogenesis of SIMD has been widely recognized. When the body is infected by pathogens, inflammasome is activated, triggering the release of pro-inflammatory cytokines and immune response. Qiu et al. Qiu et al. (2019) reveals that LPS could increase the sensitivity of H9c2 cells to high glucose and hypoxia/reoxygenation and aggravated high glucose and hypoxia/reoxygenation-induced H9c2 cell injury by promoting ROS production to induce NLRP3 inflammasome-mediated pyroptosis. In LPS-treated mice and LPS-treated H9c2 cells, it has been found that LPS stimulation promotes the translocation of NLRP3 from nucleus to cytoplasm and dissociation of TXNIP from thioredoxin (Li et al., 2019d). Yang et al. Yang et al. (2019) find that knock-down of TXNIP inhibits LPS-induced inflammasome activation. The knockout of NLRP3 alleviates cardiac atrophy and cardiomyopathy in CLP mice and therefore ameliorates SIMD (Busch et al., 2021).

## 5 MERCS as a drug target

As mentioned above, MERCs play an important role in SIMDs. Therefore, to find or develop novel potential therapeutic agents for regulating the formation of MERCs may be a promising treatment for SIMD. We agree with Magalhaes Rebelo and colleagues that drugs could affect MERCs from three levels (Magalhaes Rebelo et al., 2020): 1) drugs that interact directly with MERCs proteins; 2) drugs that affect the expression of MERCs proteins; 3) drugs that affect the upstream signaling pathway of MERCs proteins. Next, we will introduce some drugs that may regulate the formation of MERCs. For more information about MERCs drugs, please refer to the studies of Magalhães Rebelo et al. Magalhaes Rebelo et al. (2020) and Dentoni et al. Dentoni et al. (2022b).

It has been reported that natural products Xestospongins B and C bind to IP3R and affect some MERCs functions (e.g., calcium signaling, autophagy, and ER stress, etc.) (Wang et al., 2019; Castro-Sepulveda et al., 2021). Furthermore, 2-aminoethyldiphenyl borate is also found to increase neuronal excitability *via* binding to IP3R (Hagenston et al., 2009). The function of IP3R can also be affected by FDA approved drugs, such as trifluoperazine (Kang et al., 2017). In addition, a number of compounds, such as aspirin (Tewari et al., 2017), Itraconazole (Head et al., 2015), Allopregnanolone (Cheng et al., 2019), have been reported to target VDAC1 and modulate its activity. Mfn2, a MERCs protein localized in both ER and mitochondria, is involved in autophagy, mitochondrial dynamics, ER stress and so on, so it is an important target for regulating MERCs. Franco et al. Franco et al. (2016) and Rocha et al. Rocha et al. (2018) develop minipeptide and small-molecule mimics targeting Mfn2 to ameliorate mitochondrial damage in CMT2A disease.

The natural compound resveratrol has been found to increase Mfn2 levels in patients with Alzheimer’s Disease (Robb et al., 2017) and induce Ca^2+^ transport from ER to the mitochondria (Madreiter-Sokolowski et al., 2016). In contrast, nicotine reduces Mfn2 levels and induces mitochondrial dysfunction and apoptosis (Hirata et al., 2016). In addition to that, studies show that crude flavonoid extract from *ErigeronErigeron* breviscapus (named breviscapine) (Bao et al., 2018) and the glucoside salidroside (Zhuang et al., 2017) also have an effect on Mfn2 levels. Metformin stabilizes the MERCs structure in insulin-resistant mice induced by the high fat and high sucrose diet, which may be associated with inhibiting the expression of VDAC1 and PACS2 and increasing Mfn2 levels (Foretz et al., 2014; Sanchez-Rangel and Inzucchi, 2017). Studies have shown that some miRNAs can target MERCs proteins. Wang et al. Wang et al. (2012) find that miR-484 could reduce the expression of Fis1 and reduce mitochondrial fission as well as apoptosis caused by hypoxia, suggesting that miR-484 mimics could regulate MERCs to improve mitochondrial function and inhibit apoptosis. AMPK is a sensor of the energetic status of the cell that regulates ATP production and affects protein function. Quercetin is considered to be a regulator of AMPK activity, in particular, it indirectly affects the activity or expression of MERCs protein TXNIP *via* AMPK (Shaked et al., 2011; Wu et al., 2013). Lithium, a treatment agent for bipolar disorder, has been found to indirectly affect autophagy proteins expression by regulating the expression of KLF4 and PARKRG (Rivera and Butt, 2019).

## 6 Conclusion

To date, numerous studies have described the structure and function of MERCs, which are involved in the regulation of various cell biological functions, including calcium signaling, lipid synthesis and transport, autophagy, mitochondrial dynamics, and ER stress. Increasing evidence suggests that MERCs-associated calcium signaling, autophagy, mitochondrial dynamics, and ER stress play important roles in SIMD, but the role of MERCs as a subcellular organelle has not yet received much attention.

Currently, there are many questions about the structure and composition of MERCs and its role in SIMD, and there is much work remains in this area. Firstly, the extraction and detection of MERCs is the basis of research on the structure and function of MERCs. It is especially important to optimize the extraction or detection method of MERCs, which makes the detection of MERCs more accurate and convenient. Secondly, more studies are needed to clarify the changes and functions of MERCs in SIMD, as well as the key biological functions or targets. Thirdly, proteins enriched in MERCs are also commonly present in the bulk ER and mitochondria, playing a variety of physiological functions. Targeting proteins in MERCs may produce off-target effects. Therefore, appropriate targets and drugs should be searched and developed, as well as suitable doses and way of drug administration, so as to moderately regulate MERCs and prevent adverse effects from increasing many or decreasing very few MERCs-associated functions.

In this review, we summarize the biological functions of MERCs and the roles of MERCs molecular components in SIMD. MERCs are expected to be the effective targets for the treatment of SIMD in the future.
